# Liposome-Coupled Antigens Are Internalized by Antigen-Presenting Cells via Pinocytosis and Cross-Presented to CD8^+^ T Cells

**DOI:** 10.1371/journal.pone.0015225

**Published:** 2010-12-17

**Authors:** Yuriko Tanaka, Maiko Taneichi, Michiyuki Kasai, Terutaka Kakiuchi, Tetsuya Uchida

**Affiliations:** 1 Department of Immunology, Toho University School of Medicine, Tokyo, Japan; 2 Department of Safety Research on Blood and Biological Products, National Institute of Infectious Diseases, Tokyo, Japan; New York University, United States of America

## Abstract

We have previously demonstrated that antigens chemically coupled to the surface of liposomes consisting of unsaturated fatty acids were cross-presented by antigen-presenting cells (APCs) to CD8^+^ T cells, and that this process resulted in the induction of antigen-specific cytotoxic T lymphocytes. In the present study, the mechanism by which the liposome-coupled antigens were cross-presented to CD8^+^ T cells by APCs was investigated. Confocal laser scanning microscopic analysis demonstrated that antigens coupled to the surface of unsaturated-fatty-acid-based liposomes received processing at both MHC class I and class II compartments, while most of the antigens coupled to the surface of saturated-fatty-acid-based liposomes received processing at the class II compartment. In addition, flow cytometric analysis demonstrated that antigens coupled to the surface of unsaturated-fatty-acid-liposomes were taken up by APCs even in a 4°C environment; this was not true of saturated-fatty-acid-liposomes. When two kinds of inhibitors, dimethylamiloride (DMA) and cytochalasin B, which inhibit pinocytosis and phagocytosis by APCs, respectively, were added to the culture of APCs prior to the antigen pulse, DMA but not cytochalasin B significantly reduced uptake of liposome-coupled antigens. Further analysis of intracellular trafficking of liposomal antigens using confocal laser scanning microscopy revealed that a portion of liposome-coupled antigens taken up by APCs were delivered to the lysosome compartment. In agreement with the reduction of antigen uptake by APCs, antigen presentation by APCs was significantly inhibited by DMA, and resulted in the reduction of IFN-γ production by antigen-specific CD8^+^ T cells. These results suggest that antigens coupled to the surface of liposomes consisting of unsaturated fatty acids might be pinocytosed by APCs, loaded onto the class I MHC processing pathway, and presented to CD8^+^ T cells. Thus, these liposome-coupled antigens are expected to be applicable for the development of vaccines that induce cellular immunity.

## Introduction

Vaccines have played an important role in disease prevention and have made a substantial contribution to public health. Upon natural infection, it is known that the host responds by inducing both humoral and cellular immunity against the pathogen. However, most of the currently approved vaccines work by inducing humoral immunity [1]–[3]. For protection against viruses that are highly mutable and frequently escape from antibody-mediated immunity, such as influenza A viruses, HIV, and HCV, humoral immunity is insufficient [4]–[7]. Consequently, the development of vaccines that induce cellular immunity is critical to novel vaccine strategies.

T lymphocytes respond to peptide fragments of protein antigens that are displayed by MHC molecules on antigen-presenting cells (APCs). In general, extracellular antigens are presented via MHC class II molecules to CD4^+^ T cells while intracellular antigens are presented via MHC class I molecules to CD8^+^ T cells [8], [9]. However, a number of reports have demonstrated that a significant level of crossover, so-called ‘cross-presentation’, occurs in APCs [10]–[14]. Using this phenomenon, novel vaccine preparation inducing antigen-specific CTLs that effectively eliminate virus-infected cells is expected. The mechanisms of cross-presentation have been studied intensively [15]–[17] while the details have been left unclear. Part of the antigens taken via phagocytosis by APCs are known to be translocated into the cytosol and degraded by local proteases [18], [19]. In another pathway, some antigens internalized into endocytic compartments are loaded onto MHC class I molecules [20].

We previously reported that antigens chemically coupled to the surface of liposomes induced antigen-specific IgG but not IgE antibody production [21], [22]. In addition, antigens chemically coupled to the surface of liposomes consisting of unsaturated fatty acids were presented not only to CD4^+^- but also to CD8^+^ T cells by APCs [23]. Since liposome-coupled antigens induce antiviral immunity [24], [25], they are expected to be applicable for the development of viral vaccines without inducing antigen-specific IgEs, which cause allergic reactions. In the present study, we investigated the mechanism by which the liposome-coupled antigens were cross-presented by APCs to CD8^+^ T cells.

## Results

### Confocal laser scanning microscopic analysis of macrophages co-cultured with DQ-OVA-liposome conjugates

**Figure 1:**
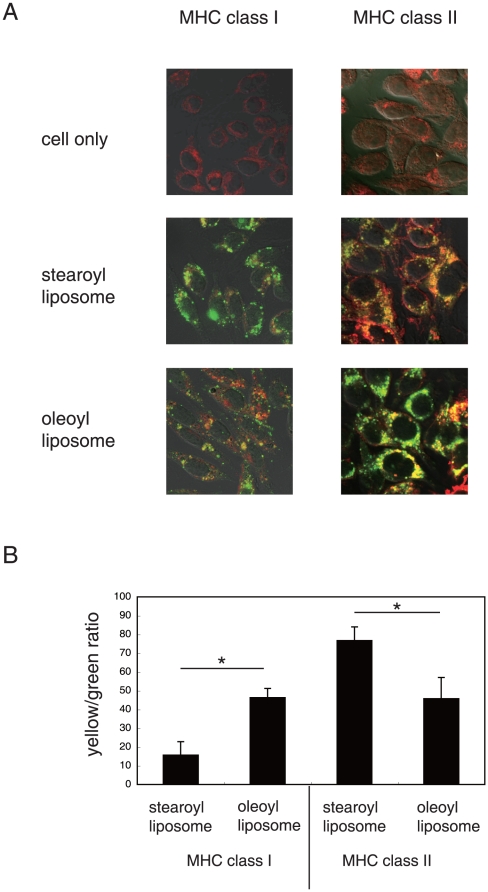
Confocal laser scanning microscopic analysis of macrophages co-cultured with DQ-OVA-liposome conjugates. A, DQ-OVA was coupled to either stearoyl or oleoyl liposomes and added to the culture of cloned macrophages expressing DM-DsRed (class II) or labeled with red fluorescein (class I), as described in Materials and Methods. Two hours after the onset of the culture, macrophages were recovered and analyzed using confocal laser scanning microscopy. These optically merged images are representative of most cells examined by confocal microscopy. Yellow, co-localization of green (DQ-OVA after proteolytic degradation) and red (macrophage DM or class I); cell only, macrophages without co-culture with DQ-OVA-coupled liposomes. B, the green- and yellow-color compartments in the immunofluorescent pictures were quantified by the image analysis software MetaMorph, as described in Materials and Methods. Ratios of the yellow to green compartments are shown. Data represent the mean values ± SD of the images shown in Fig. 1A. Asterisk, significant (*p*<0.01) difference of samples.

MHC class I of macrophages were stained with red fluorescein-labeled anti-mouse H-2D^d^ mAb (Fig. 1A: left column), and MHC class II of macrophages were labeled with DM-DsRed (Fig. 1A: right column) as described in Materials and Methods. DQ-OVA, which exhibits green fluorescein upon proteolytic degradation, was coupled to liposomes consisting of unsaturated (oleoyl) or saturated (stearoyl) fatty acid, and added to the culture of macrophages. After incubation for 2 hr, the recovered macrophages were analyzed using confocal laser scanning microscopy. The results shown in Fig. 1 demonstrate that DQ-OVA coupled to oleoyl liposomes was processed at both MHC class I and class II compartments, while most of the DQ-OVA coupled to stearoyl liposomes was processed at the MHC class II compartment.

### Differential manner of internalization by APCs of antigens coupled to liposomes with two kinds of lipid

**Figure 2:**
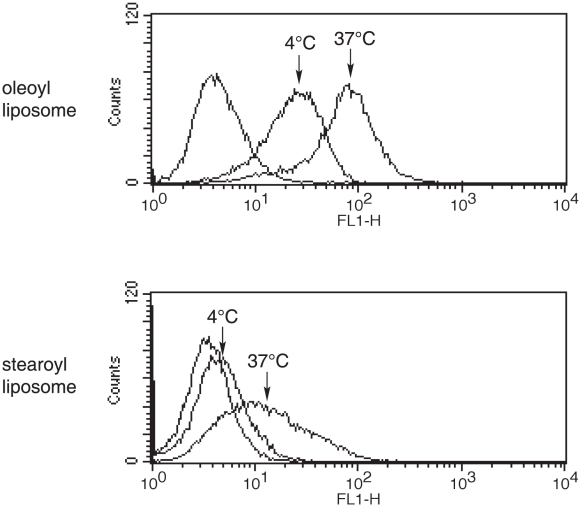
Uptake of liposome-coupled OVA by macrophages. Alexa-labeled OVA was coupled to either stearoyl or oleoyl liposomes and added to the culture of cloned macrophages as described in Materials and Methods. Thirty minutes after the onset of the culture, macrophages were recovered and analyzed using flow cytometry.

Alexa_488_-labeled OVA were coupled to liposomes and were added to the cultures of macrophages. As shown in Fig. 2, OVA coupled to oleoyl liposomes were internalized by APCs more efficiently than those coupled to stearoyl liposomes at 37°C. Interestingly, OVA coupled to oleoyl liposomes but not stearoyl liposomes were internalized significantly by APCs even in a 4°C environment.

### Effect of inhibitors on uptake of liposome-coupled antigens by APCs

**Figure 3:**
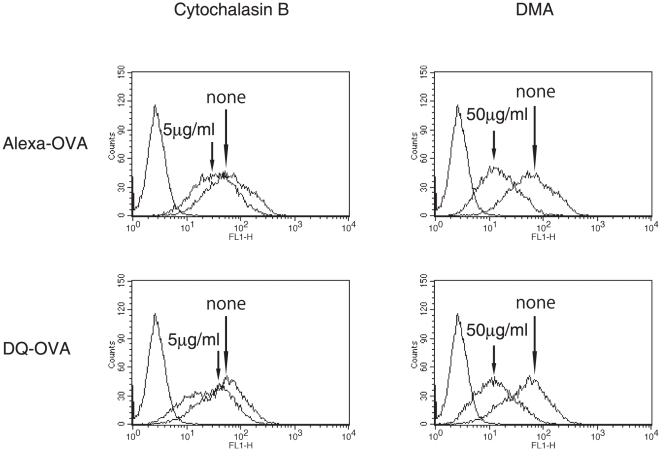
Influence of inhibitors for uptake of OVA coupled to oleoyl liposomes by macrophages. Alexa- or DQ-labeled OVA was coupled to oleoyl liposomes and added to the culture of macrophages as described in Materials and Methods. Treatment of macrophages with cytochalasin B or DMA was done 60 minutes prior to the addition of OVA-liposome conjugates.

One of two kinds of inhibitors, cytochalasin B and DMA, which inhibit APC phagocytosis and pinocytosis of antigens, respectively, was added to the culture of macrophages 1 hr prior to the addition of Alexa_488_-OVA- or DQ-OVA-coupled oleoyl liposomes. One hour later, flow cytometric analysis was performed. As shown in Fig. 3, the effect of cytochalasin B on the antigen uptake and digestion of liposome-coupled OVA by APCs was limited. On the other hand, DMA significantly reduced both antigen uptake and digestion of antigens by macrophages.

### Localization of antigens coupled to liposomes in APCs

**Figure 4:**
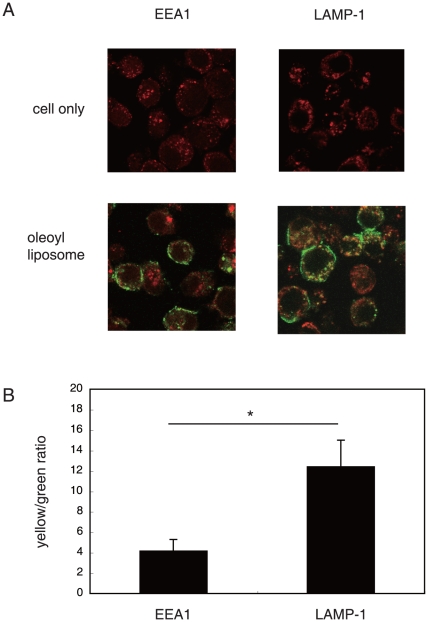
Intracellular localization of liposomal antigens taken up by macrophages. A, DQ-OVA was coupled to oleoyl liposomes and added to the culture of cloned macrophages of which endosomal marker EEA1-positive compartments, or lysosomal marker LAMP-1-positive compartments were stained as described in Materials and Methods. Two hours after the onset of the culture, macrophages were recovered and analyzed using confocal laser scanning microscopy. These optically merged images are representative of most cells examined by confocal microscopy. Yellow, co-localization of green (DQ-OVA after proteolytic degradation) and red (macrophage EEA1 or LAMP-1); cell only, macrophages without co-culture with DQ-OVA liposomes. B, the green- and yellow-color compartments in the immunofluorescent pictures were quantified by the image analysis software MetaMorph, as described in Materials and Methods. Ratios of the yellow to green compartments are shown. Data represent the mean values ± SD of the images shown in Fig, 4A. Asterisk, significant (*p*<0.01) difference of samples.

DQ-OVA-coupled oleoyl liposomes were added to the culture of macrophages in which either EEA1 or LAMP-1 were co-stained. The co-localization of the liposome-coupled antigens and intracellular organelles in the APCs was analyzed using confocal laser scanning microscopy. As shown in Figure 4, although most of the DQ-OVA coupled to oleoyl liposomes was processed beyond LAMP-1-expressing compartments (green spots), a portion of DQ-OVA was processed at compartments expressing LAMP-1 (yellow spots). Co-localization of EEA1-expressing compartments with liposome-coupled-DQ-OVA was significantly less than that of LAMP-1-expressing compartments with DQ-OVA (Fig. 4B).

### T cell activation by APCs pulsed with liposomal antigen

**Figure 5:**
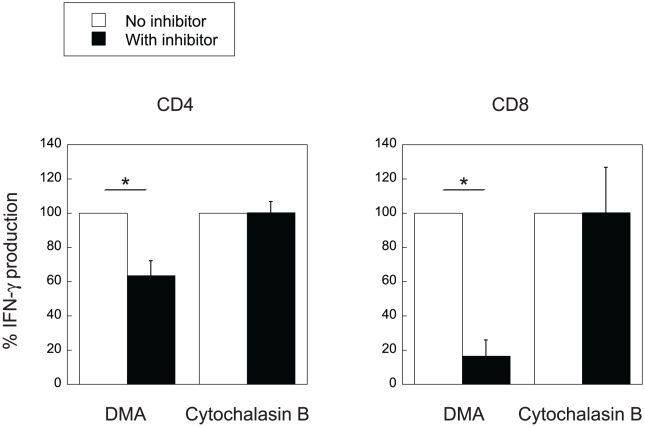
IFN-γ production by splenic CD4/CD8^+^ T cells of mice immunized with OVA after co-culture with CD11c^+^ cells pulsed with OVA coupled to oleoyl liposomes. Splenic CD4/CD8^+^ T cells were taken from mice immunized with OVA and were cultured with CD11c^+^ cells pulsed with OVA coupled to oleoyl liposomes with or without inhibitors as described in Materials and Methods. IFN-γ production of T cells in the supernatants in the absence of inhibitors was normalized to 100%. Data represent the mean values ± SD of triplicate culture. Asterisk, significant (*p*<0.01) difference as compared with the ‘no inhibitor’ group.

In agreement with the results shown in Fig. 3, antigen presentation by APCs pulsed with liposomal antigen was significantly inhibited by DMA but not by cytochalasin B in both CD4^+^- and CD8^+^ T cell responses (Fig. 5).

## Discussion

In general, extracellular antigens are presented via MHC class II molecules to CD4^+^ T cells, whereas intracellular antigens are presented via MHC class I molecules to CD8^+^ T cells. Consequently, most APCs do not present exogenous antigens via MHC class I since exogenous antigens do not gain access to the cytosolic compartment. Therefore, exogenous antigens usually do not prime CTL responses *in vivo*. This segregation of exogenous antigens from the class I pathway is important to prevent CTL from killing healthy cells that have been exposed to foreign antigens but are not infected [26]. However, there are several exceptions to this rule, reflecting the ability of the exogenous antigens to be delivered into the cytosolic compartments [13]–[17].

We have previously reported that antigens coupled to the surface of liposomes comprised of unsaturated fatty acid are presented to both CD4^+^- and CD8^+^ T cells [23]. Confocal laser scanning microscopic analysis demonstrated that a portion of the liposome-coupled antigens were taken up and processed beyond the MHC class II compartment. In the present study, we confirmed that OVA coupled to oleoyl liposomes was processed at both the MHC class I and class II compartments (Fig. 1). Flow cytometric analysis demonstrated that OVA coupled to oleoyl liposomes was incorporated more efficiently by macrophages than OVA coupled to stearoyl liposomes (Fig. 2). Furthermore, OVA coupled to oleoyl liposomes was taken up by macrophages even in a 4°C environment, in which antigen entry could only occur via plasma membrane translocation. In general, antigen processing pathways largely depend on the route of antigen uptake, and liposomes with a certain lipid component are known to fuse with the plasma membrane [27]. The uptake of OVA coupled to oleoyl liposomes in a 4°C environment observed in the present study suggested that oleoyl liposome might fuse with the plasma membrane and thereby allow the liposome-coupled antigen direct access to the cytosol. The role of endocytosis in the uptake of the liposomal antigen was further examined by using specific inhibitors for antigen uptake (Fig. 3). Cytochalasin B treatment of APCs prior to the addition of liposomal antigen in the culture had little effect. However, treatment of APCs with DMA significantly reduced the uptake of liposome-coupled OVA. Consequently, it was suggested that antigens coupled to oleoyl liposomes might be taken up by APCs via at least two pathways, penetration and pinocytosis. The analysis of intracellular pathways of antigens coupled to oleoyl liposomes using confocal laser scanning microscopy demonstrated that a portion of liposomal antigens taken up by APC were translocated to the lysosomal compartments expressing LAMP-1 (Fig. 4), suggesting that the liposomal antigens processed at lysosomal compartment and beyond lysosomal compartment might be presented to CD4/CD8^+^ T cells via MHC class II and class I, respectively. In agreement with the results of antigen uptake shown in Fig. 3, the treatment of splenic CD11c^+^ cells with DMA significantly reduced antigen presentation of liposomal antigens to both CD4^+^- and CD8^+^ T cells as evaluated by T-cell activation (Fig. 5).

It was reported that pinocytosis and scavenger receptor-mediated endocytosis by APC facilitate antigen presentation to CD4^+^ T cells; by contrast, mannose receptor-mediated endocytosis by APC has been shown to facilitate antigen presentation to CD8^+^ T cells [28]. However, as described in Materials and Methods, the oleoyl liposomes used in the present study do not contain mannose.

Thus, the data in the present study demonstrated that antigens coupled to oleoyl liposomes were internalized by APCs through both penetration and pinocytosis. The antigens coupled to the surface of oleoyl liposomes were processed at both MHC class I and class II compartments and presented to CD4^+^- and CD8^+^ T cells. Although the detailed pathway leading to presentation to both CD4^+^- and CD8^+^ T cells remains unclear, the observed behavior of antigens coupled to oleoyl liposome in APCs seems quite unique. Taken together, coupling of antigens to oleoyl liposome might potentially serve as a novel method to induce both humoral and cellular immunity.

## Materials and Methods

### Mice

CBF1 mice (8 weeks of age, female) were purchased from SLC (Shizuoka, Japan). All experiments were approved (No. 208021 and 209082) by an independent animal ethics committee at National Institute of Infectious Diseases, Tokyo, Japan.

### Chemicals

All phospholipids were provided by NOF Co. (Tokyo, Japan). Reagent grades of cholesterol were purchased from Wako Pure Chemical (Osaka, Japan).

### Antigens and reagents

Ovalbumin (OVA, Grade VII) was purchased from Sigma-Aldrich. For the analysis of the processing of liposome-coupled OVA by macrophages, DQ-OVA, which exhibits green fluorescence upon proteolytic degradation, was purchased from Molecular Probes, Inc. Synthetic CpG ODN (5002: TCCATGACGTTCTTGATGTT) was purchased from Invitrogen and was phosphorothioate-protected to avoid nuclease-dependent degradation.

### Fluorescence labeling of OVA

OVA was labeled with fluorescence using an AlexaFluor 488 protein labeling kit (Invitrogen) according to the manufacturer's protocol.

### Liposomes

Liposomes consisting of two different kinds of lipid were used in this study. Liposomes consisting of saturated fatty acids were composed of distearoyl phosphatidylcholine, distearoyl phosphatidyl ethanolamine, distearoyl phosphatidyl glycerol acid, and cholesterol in a 4∶3∶2∶7 molar ratio (stearoyl liposomes), and liposomes consisting of unsaturated fatty acids were composed of dioleoyl phosphatidylcholine, dioleoyl phosphatidyl ethanolamine, dioleoyl phosphatidyl glycerol acid, and cholesterol in a 4∶3∶2∶7 molar ratio (oleoyl liposomes). The crude liposome solution was passed through a membrane filter (nucleopore polycarbonate filter, Coster) with a pore size of 0.2 mm.

### Coupling of OVA to liposomes

Liposomal conjugates with plain OVA, Alexa-labeled OVA, or DQ-OVA were prepared essentially in the same way as described previously [22]. Briefly, to a mixture of 90 mg of liposomes and 6 mg of OVA in 2.5 ml phosphate buffer (pH 7.2), 0.5 ml of 2.5% glutaraldehyde solution was added in dropwise fashion. The mixture was stirred gently for 1 h at 37°C, and then 0.5 ml of 3 M glycine-NaOH (pH 7.2) was added to block excess aldehyde groups. This was followed by incubation overnight at 4°C. The liposome-coupled OVA and uncoupled OVA in the resulting solution were separated using CL-4B column chromatography (Pharmacia). The amount of lipid in the liposomal fraction was measured using a phospholipid content assay kit (Wako Pure Chemical). The OVA-liposome solution was adjusted to 10 mg lipid/ml in PBS, sterile-filtered using a Millex-HA syringe filter unit (0.45 µm, Millipore), and kept at 4°C until use.

### Quantification of OVA coupled to liposome

For the measurement of OVA coupled to liposome, radio-labeled OVA (*methyl*-^14^C; purchased from New England Nuclear) was mixed with cold OVA and used for coupling with liposome and for determining the calibration curve. The radioactivity of the resulting OVA-liposome solution was counted using a calibration curve. The amounts of OVA coupled to stearoyl and oleoyl liposomes were 47.0 and 46.8 µg/mg lipid, respectively.

### Immunization

Mice were immunized subcutaneously (s.c.) with the OVA-liposome conjugate at a dose of 1 mg lipid/100 µl/mouse in the presence of 5 µg/mouse CpG.

### Cloned macrophage hybridoma

Macrophage hybridoma clone 39, obtained from the fusion of splenic adherent cells from CKB mice and P388D1 [29], was used.

### Construction and expression of the fusion protein, DM-DsRed, in macrophage clone 39

The DNA fragment coding the full-length H2-DMβ2 [30] was amplified by PCR with two primers (5′-ATGGCTGCACTCTGGCTGCTGCTGCTGGT-3′ and 5′-GATGCCGTCCTTCTGGGTAGGTGGATCC-3′). The PCR product was cloned into the CMV promoter-driven expression plasmid pDsRedN1 (BD Clontech). This construct omitted the stop codon of H2-DMβ2 and encoded the H2-DMβ2 fused with DsRed. The cloned plasmid DNA was transfected to macrophage hybridoma clone 39 with Effectene transfection reagent (Qiagen) according to the manufacturer's protocol. During the transfection to clone 39, the medium containing cDNA and the transfection reagent was replaced with fresh medium after an 8-h transfection, and then clone 39 was cultured for 40 h. To obtain stable cell lines, clone 39 was passaged at 1∶5 into RPMI 1640 containing 10% FCS with 50 µg/ml geneticin (G-418; Sigma-Aldrich). Cells showing the best fluorescence were selected using a FACS Vantage cell sorter (BD Bioscience). After cell sorting, clone 39 expressing DM-DsRed was cultured in RPMI 1640 containing 10% FCS with 200 µg/ml geneticin.

### Flow cytometry

To investigate the capture of OVA-liposome conjugates by macrophages, macrophage clone 39 was incubated for 30 min at 4°C or 37°C in the presence of fluorescence-labeled OVA-liposome conjugates that contained a final concentration of 4 µg/ml OVA. After the incubation, cells were washed with ice-cold PBS. In the case of using Alexa-labeled OVA-liposome conjugates, cells were then incubated with 1.2 µg/ml trypan-blue for 5 min at 4°C to block the fluorescence of Alexa-OVA attached to the cell surface. After the cells were washed, they were analyzed on a FACS Caliber flow cytometer (BD Bioscience). The histograms of fluorescence distribution were plotted as the number of cells versus fluorescence intensity on a logarithmic scale.

### Confocal laser scanning microscopy

To investigate the localization of OVA-liposome conjugates by macrophages, macrophage clone 39 or DM-DsRed-expressing cloned macrophage 39 was cultured for 18 h at 37°C on 8-hole heavy Teflon-coated slides (Bokusui Brown) and was then incubated with DQ-OVA-liposome conjugates, prepared using oleoyl or stearoyl liposomes, for 2 h at 37°C. The slides were then washed with MEM and fixed with 4% paraformaldehyde in PBS for 10 min at room temperature. After fixation, they were incubated for 10 min in 0.1 M glycine-HCl (pH 7.0) to block the remaining aldehyde residue. They were then washed two times in PBS. After washing, the slides were sealed with PBS:glycerin (1∶9) and analyzed under an LSM510 confocal laser scanning microscope system (Zeiss). For analysis of co-localization of OVA and MHC class I, early endosomal antigen 1 (EEA1) or lysosomal-associated membrane protein-1 (LAMP-1) after blocking of the remaining aldehyde residue, cloned macrophage 39 was subsequently permeabilized with 0.05% saponin-TBS for 10 min at room temperature. After being washed twice with PBS, they were reacted with biotin-conjugated mouse anti-mouse H-2D^d^ mAb (34-2-12, 10 µg/ml; BD Biosciences), goat anti-mouse EEA1 polyclonal antibody (N19, 1 µg/ml; Santa Cruz Biotechnology) or rat anti-mouse LAMP-1 monoclonal antibody (1D4B, 1 µg/ml; Santa Cruz Biotechnology) for 18 h at 4°C. After being washed three times with TBS, they were reacted with Alexa 546-conjugated streptavidin (1∶200 diluted; Invitorogen) to detect MHC class I, Alexa Fluor 568-labeled Ab (rabbit anti-goat IgG, 10 µg/ml; Invitrogen) to detect EEA1 or Alexa Fluor 568-labeled Ab (goat anti-rat IgG, 10 µg/ml; Invitrogen) to detect LAMP-1 for 4 h at room temperature. They were then washed two times in TBS. After the washing, the slides were sealed with PBS:glycerin (1∶9) and analyzed under an LSM510 confocal laser scanning microscope system (Zeiss).

### Quantification of immunofluorescent pictures and statistics

Quantification of confocal image analysis was done by single cell identification using the image analysis software MetaMorph (Molecular Devices Co., Tokyo, Japan), and the relative fluorescence intensity of green, red, and yellow pixels was assessed. The relative fluorescence intensity of all individual colors was then expressed as percent of the total fluorescence intensity. *p* values were calculated by the Student's t test with two-tailed distribution and two-sample unequal variance parameters.

### Inhibition Studies of Antigen Uptake

In the case of inhibition studies, cloned macrophage 39 or CD11c^+^ cells were incubated with indicated inhibitors 60 min before and throughout the antigen pulse. Cytochalasin B [31] and DMA [28] were purchased from Sigma.

### Preparation of CD11c^+^ cells and CD4^+^- and CD8^+^ T cells

CD11c^+^ spleen cells of naïve mice and CD4^+^ T and CD8^+^ T spleen cells of mice immunized with OVA-liposome conjugates were prepared with the magnetic cell sorter system MACS, according to the manufacturer's protocol using anti-CD11c, anti-CD4 and anti-CD8 antibody-coated microbeads (Miltenyi Biotec).

### Culture of CD4^+^- and CD8^+^ T cells with CD11c^+^ cells pulsed with OVA

CD11c^+^ cells were incubated with or without the indicated inhibitors for 60 min in a 24-well plate prior to the addition of OVA-liposome conjugates made using oleoyl liposomes. The final concentration of OVA-liposome added to the macrophage culture was 500 µg lipid/ml, which included 24 µg OVA. After 60 minutes' incubation, CD11c^+^ cells were washed 3 times in ice-cold medium and 2×10^5^ cells were co-cultured with 5×10^5^ CD4^+^ T cells or CD8^+^ T cells, in a 48-well plate. A preliminary experiment showed that the optimal culture period in the above culture condition was 2 days for IFN-γ production by CD4^+^ T cells and 5 days for IFN-γ production by CD8^+^ T cells. After incubation in a CO_2_ incubator for 2 or 5 days, the culture supernatants were collected and assayed for IFN-γ.

### IFN-γ Assay

IFN-γ in the culture supernatants was measured using the Biotrak mouse ELISA system (GE Healthcare). All test samples were assayed in duplicate, and the SD in each test was always <5% of the mean value.
